# Lateral periodontal cyst: A rare clinicopathological presentation mimicking a residual cyst

**DOI:** 10.4317/jced.58668

**Published:** 2022-01-01

**Authors:** Davi-da Silva Barbirato, Mariana-Fampa Fogacci, Monique-Oliveira Rodrigues, Belmiro-Cavalcanti-do Egito Vasconcelos, Maria-Cynésia-Medeiros de Barros, Fábio-Ramôa Pires

**Affiliations:** 1 Postdoctoral fellow, DDS, PhD. Dental School, University of Pernambuco, Recife, PE, Brazil; 2 DDS, PhD. Adjunct Professor, Department of Clinical and Preventive Dentistry, Federal University of Pernambuco, Recife, PE, Brazil; 3 DDS, PhD. Professor of Periodontics, Graduation and Post-graduation in Dentistry/Periodontics, Federal University of Rio de Janeiro, Rio de Janeiro, RJ, Brazil; 4 DDS, PhD. Associate Professor, Department of Oral and Maxillofacial Surgery, University of Pernambuco, Recife, PE, Brazil/Staff of the Oral and Maxillofacial Surgery service, Hospital da Restauração, Recife, PE, Brazil; 5 DDS, PhD. Professor, Department of Oral Pathology, Laboratory of Oral Pathology, School of Dentistry, State University of Rio de Janeiro, Rio de Janeiro, Brazil

## Abstract

This article describes an unusual clinical-radiographic presentation of a lateral periodontal cyst, as a differential diagnosis of a residual cyst, following the ‘CARE guidelines for case reports’. The radiolucent lesion was identified on the imaging exam of a 53-year-old male patient. Based on radiographic findings and aspiration puncture, the probable diagnosis was a residual cyst; however, histological analysis revealed a thin, non-inflamed fibrous capsule covered by some epithelial layers in most of the lesion. The definitive diagnosis was a lateral periodontal cyst with unusual clinical and radiographic features. The cyst was surgically enucleated and local bone neoformation was observed, with no signs of recurrence after 12 months. The results of this study suggest that a radiolucent lesion, suggestive of a residual cyst or keratocyst in the maxilla, may correspond to a lateral periodontal cyst. In this context, the histopathological analysis of the cyst is essential for the definitive diagnosis.

** Key words:**Cysts, odontogenic cysts, periapical cysts, periodontal cysts.

## Introduction

The lateral periodontal cyst (LPC) is an uncommon entity, representing 0,4% to 0,7% of all oral and maxillofacial cysts and 0,6% to 2% of the odontogenic cysts (1-6). It is considered a non-inflammatory developmental cyst associated with the proliferation of odontogenic remnants, possibly from the dental lamina, reduced enamel epithelium or epithelial rests of Malassez (2,6-9). It is characterized by a unilocular radiolucency between the roots of vital erupted teeth, mostly premolars, canines and incisives (2,6,7,9-12). Most cases affect adults in their 5th to 7th decades of life, with no gender or a slight male predilection, and about 70% of the cases are located in the mandible (2,4,6,7,9,11-14). When presenting the typical clinical and radiological features, the main differential diagnosis for LPC include lateral periapical cyst, odontogenic keratocyst and other uncommon odontogenic tumors (such as squamous odontogenic tumor) (2,15). Some LPC, however, can present as multilocular radiolucencies (in a multicystic pattern so-called “botrioid odontogenic cyst”) (2,7), close to a periapical location (16,17), mimicking a dentigerous cyst (18), or resembling periodontal defects (19), and in these specific situations differential diagnosis can be broader. Although the clinical and radiological features of typical LPC are very suggestive, final diagnosis depend upon histological analysis of the surgical specimen (2,10,15).

In very rare instances LPC can mimic the clinical and radiological features of residual cysts, turning differential diagnosis a challenging issue. In these specific cases, LPC is usually not considered in differential diagnosis (20). So, the aim of the present study is to report a case of a LPC clinically and radiologically resembling a residual cyst, calling attention to the importance of also including this entity in the differential diagnosis.

## Case Report

**Figure 1:**
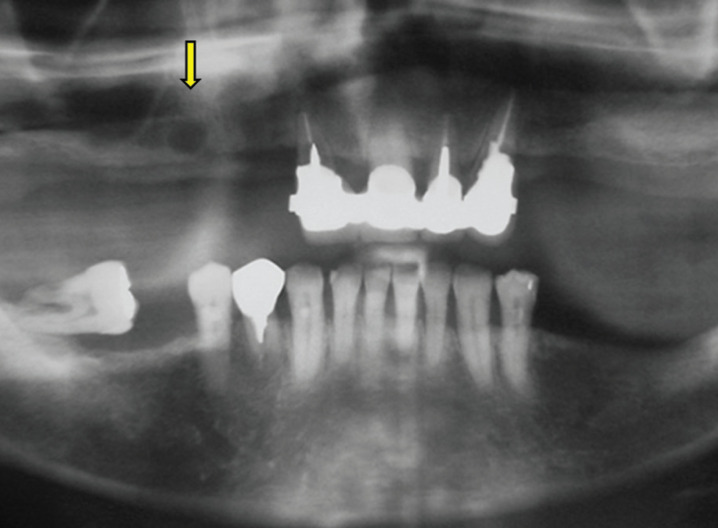
Panoramic radiograph showing an unilocular radiolucency in the right maxilla (arrow).

**Figure 2:**
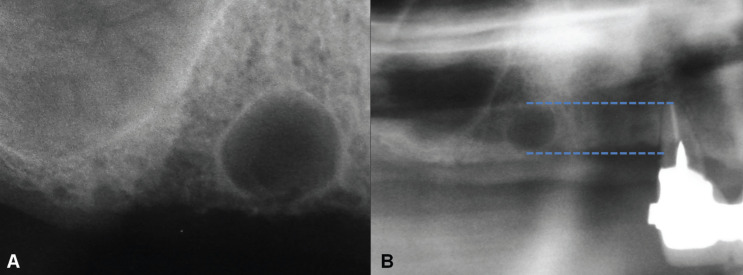
A. Periapical radiograph showing a well-defined rounded unilocular radiolucency in the upper right premolar area. B. Height relationship of the image with the apical third of the upper right lateral incisor.

A 53 year old male was referred for evaluation of an asymptomatic radiolucent image observed in the right maxilla during routine dental treatment planning. Clinical examination showed that the area was covered by normal mucosa and that there was a slight asymptomatic swelling in the buccal cortical plate in the edentulous area of upper right premolars. The patient reported that the upper right canine, premolars and molars were extracted approximately 10 years before and a partial removable appliance was placed. Panoramic and periapical radiographs showed a 1,0 cm well-defined round unilocular radiolucency in the edentulous premolar area, in close association with the superficial cortical bone (Fig. 1,2a). The radiolucent image was located at the height of the apical third of the remaining upper right lateral incisor (Fig. 2b). There were some areas compatible with fenestration of the cortical bone both in the oclusal and buccal areas due to reduction of the height of the alveolar ridge. Provisional clinical diagnosis included residual cyst and odontogenic keratocyst and the patient was scheduled for surgical removal of the lesion.

After oral antisepsis with 15 ml of 0.12 % chlorhexidine digluconate (Perioxidin®, Lacer – GlaxoSmithKline Brasil, Rio de Janeiro-RJ, Brazil) for one minute, the local anesthesia with vasoconstrictor [2 % lidocaine hydrochloride with epinephrine 1: 100,000 (Alphacaine® 100, DFL, Rio de Janeiro, Brazil)] was performed, followed by aspiration puncture with a disposable 18G needle (Becton, Dickinson and Company – BD©, New Jersey, EUA), that revealed a slightly yellowish liquid content. Due to the buccal expansion, a modified Wasmund incision (palatal approach of the horizontal incision) was performed for surgical access to the lesion. Ostectomy for access and enucleation of the whole lesion was performed with Fedi chisels and surgical detachers (Hu-Friedy® Manufacturing Co. LLC, Chicago, USA), respectively. The capsule was completely removed, and the pathological bone cavity was curetted under abundant irrigation with sterile 0.9 % sodium chloride solution for injection (Cristália©, Rio de Janeiro, Brazil). The primary closure of the mucoperiosteal flap was performed with a single interrupted suture using 4-0 non-resorbable mononylon-ethylon suture (Ethicon®, Inc., Johnson & Johnson©, New Jersey, USA).

**Figure 3:**
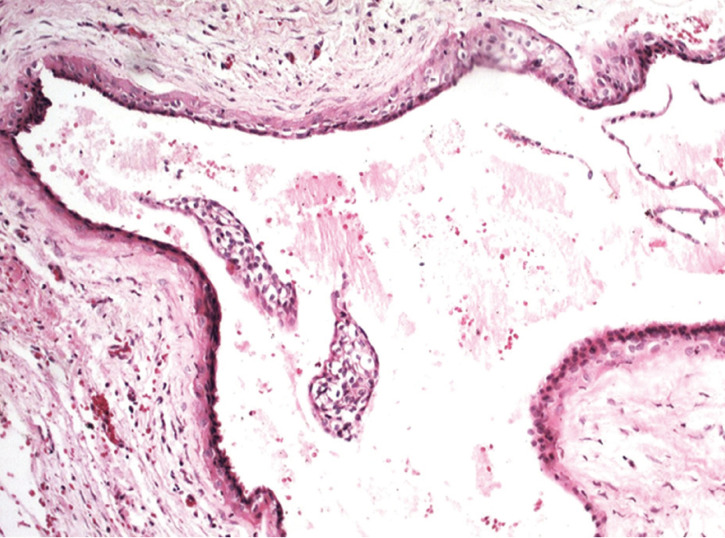
Histological features of the lateral periodontal cyst showing a cystic cavity lined by a thin stratified squamous epithelium showing focal areas of epithelial thickening and clear cells (Hematoxylin and eosin, 400x).

The surgical specimen was immersed in 10% buffered formaldehyde and submitted to conventional histological processing in the Oral Pathology Laboratory, Dental School, Rio de Janeiro State University, Rio de Janeiro-RJ, Brazil. Hematoxylin and eosin-stained five micrometers sections were analyzed under light microscopy and histological features revealed a cystic cavity lined by a thin stratified squamous epithelium showing focal areas of epithelial thickening and clear cells (Fig. 3). The adjacent fibrous connective tissue showed focal areas of chronic inflammatory infiltrate and hemorrhage. Final diagnosis was LPC.

**Figure 4:**
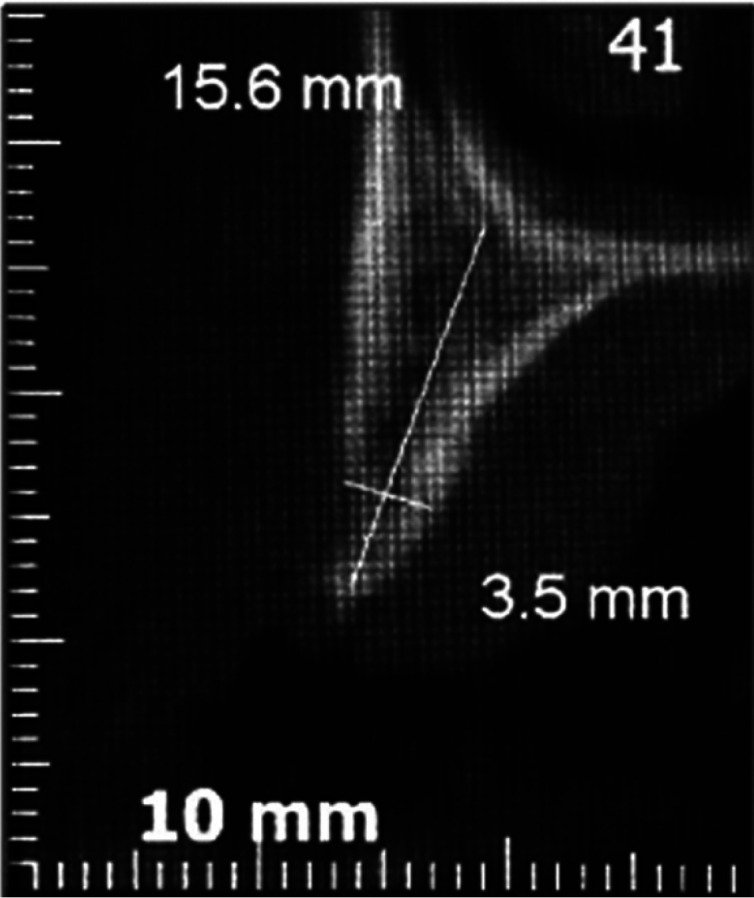
Six-month post-surgery cone beam computed tomography scan showing bone neoformation in the area.

Post-operatory period was uneventful and sutures were removed after 7 days. The patient was scheduled for periodic follow-up and a cone-beam computed tomography scan taken 6 months after the surgery showed an area of bone neoformation (Fig. 4). The patient showed no evidences of recurrence after a 1 year follow-up interval.

## Discussion

LPC is an uncommon developmental odontogenic cyst, comprising less than 2% of all odontogenic cysts (1,3-5). When presenting as a well-defined unilocular radiolucency located between the roots of two teeth in the premolar-canine-incisive area affecting an adult in the fifth or sixth decades of life, LPC is a likely provisional diagnosis (2,9,10). In these specific cases, the most common differential diagnosis is lateral periapical cyst, odontogenic keratocyst, central giant cell lesion, ameloblastoma, and squamous odontogenic tumor. Some LPC, however, do not fulfill all clinical and radiological typical features and, in these cases, considering LPC as a possible differential diagnosis can be quite challenging. Some LPC have been described in a periapical location and in these specific cases it can be very difficult to rule out the possibility of a periapical cyst (16,17). In other situations, LPC can present as larger multilocular radiolucencies, in the pattern so-called “botrioid odontogenic cyst”, and in these cases it is essential to rule out the possibility of ameloblastomas and odontogenic keratocysts (2,9).

In very rare situations, LPC can mimic the clinical and radiological features of a residual cyst, presenting as a well-defined unilocular radiolucency in an edentulous area. In these cases, alveolar bone resorption during a long period after dental extractions can cause reduction of the height of the alveolar bone and proximity of the cyst to the oral mucosal surface, as shown by the present case. Mendes & van der Waal (20) have reported a case of LPC mimicking a residual cyst in the edentulous area of the left lower first molar extracted 12 years before, discovered as an incidental finding and presenting no clinical alterations, similar to the present case. These authors have additionally reported another LPC mimicking a residual cyst in the edentulous area of the left lower premolars extracted four years earlier, presenting as a unilocular radiolucency producing rupture of the cortical bone and local bluish swelling in the area. Cohen *et al*. (11) have reported the clinicopathological features of 37 LPC and have included radiographs of two cases that resembled residual cysts. Rasmusson *et al*. (12) have reported the clinicopathological features of 32 LPC and a clinical diagnosis of residual cyst was provided in 4 cases, but no additional individual information was available. Siponen *et al*. (21) reported 4 cases of multifocal LPC and one of the cysts from case 4 radiographically resembled a residual cyst. In almost all previously reported cases/images of LPC mimicking a residual cyst it is important to call attention that the unilocular radiolucency was located close to the alveolar cortical bone and not in the most expected height for a residual cyst. In the present report, the unilocular radiolucency was located about the same height of the apical third of the nearest teeth, turning differential radiological diagnosis even more difficult. It is also important to call attention that the present case was located in the maxilla, which is affected in only about 30% of the LPC (2,9).

Histological analysis is the gold standard for the diagnosis of LPC and this cyst is characterized by an epithelial lining showing focal thickenings and clear cells. The most frequent radiological differential diagnosis for LPC, lateral periapical cyst and odontogenic keratocyst, show different histological characteristics and can be quite easily excluded from a microscopic perspective (2,11,15). Gingival cyst of the adult, another developmental odontogenic cyst, can show very similar histological features but, contrarily to LPC, it is located only in the soft tissue of the gingival or alveolar mucosa.

In conclusion, it is important for clinicians and oral surgeons to be aware of the typical and not typical clinical and radiological features associated with LPC. Although it is usually characterized as a unilocular radiolucency between the roots of erupted teeth, some rare cases, as the one included in the present report, can mimic the radiological aspects of residual cysts and should be included in their differential diagnosis.
